# Subacute phase treatment of subperiosteal hematoma of the orbit with epidural hematoma in the frontal cranial fossa: Case report

**DOI:** 10.1186/1471-2415-12-18

**Published:** 2012-06-28

**Authors:** Taro Mikami, Jiro Maegawa, Mayu Mutou Kuroda, Yasushi Yamamoto, Kazunori Yasumura

**Affiliations:** 1 Department of Plastic and Reconstructive Surgery, Yokohama City University Hospital, Fukuura 3-9, Kanazawa-ku, Yokohama, 236-0004, Japan

**Keywords:** Subperiosteal hematoma of the orbit, surgery, subacute stage, subfrontal extradural hematoma

## Abstract

**Background:**

Subperiosteal hematoma of the orbit is one of the rare lesions that cause exophthalmos after craniomaxillofacial trauma. Presently, there is no consensus for how to treat this disease. Although some reports have suggested a conservative type of therapy, others have recommended surgical treatments be done during the early stages.

**Case presentation:**

This case report provides details on the clinical course of a 9-year-old girl with subperiosteal hematoma of the orbit. In this particular patient, a rare case of ipsilateral subfrontal extradural hematoma was also observed. Due to our performing the surgical intervention during the subacute stage, functional complications as well as cosmetic problems were avoided.

**Conclusion:**

Our results demonstrate that surgical treatments for subperiosteal hematoma of the orbit should be delayed until it can be confirmed that a patient has no other complications. On the other hand, once it has been confirmed that the patient has no other existing problems, immediate surgical therapy with a small skin incision followed by the setting of a drain is recommended in order to achieve an early resolution and avoid complications.

## Background

The differential diagnosis of proptosis after craniomaxillofacial trauma includes orbital fracture, cavernous fistula, orbital emphysema, retrobulbar hematoma and orbital abscess. Subperiosteal hemangioma of the orbit, which may cause proptosis, is a rare injury. In some cases, the lesion shows diplopia, malposition of the eye, ophthalmoplegia and blepharohematoma. In the literature, there are both reports that suggest using a wait-and-see treatment with the expectation that natural extinction will occur, while others suggest that an early recovery can be attained by employing surgical therapy during the early stages.

In this report, we present the case of a 9-year-old girl with subperiosteal hematoma of the right orbit who also exhibited extradural hematoma in the frontal cranial fossa. The symptoms observed included diplopia, proptosis of the right eye and hypoophthalmia. After the patient underwent surgical treatment during the subacute stage, all of her symptoms disappeared and she completely recovered.

## Case presentation

A 9-year-old girl fell from her school’s morning assembly platform and knocked her head against the ground. Although she did not lose consciousness, she immediately noticed diplopia with right proptosis. She was examined on the same day at the Department of Ophthalmology at a nearby hospital, and underwent computed tomography (CT) of her head. After noting she had a subfrontal extradural hematoma on the right side with an ipsilateral subperiosteal hematoma in her orbit, she was admitted to the Department of Neurosurgery for further observation.

The patient was discharged 3 days after her admission, as follow-up CT indicated that neither the extradural hematoma nor the subperiosteal hematoma worsened during this time. However, since the patient showed no improvement of her right exophthalmos with diplopia, she was brought to our institution 9 days after the initial injury.

**Figure 1:**
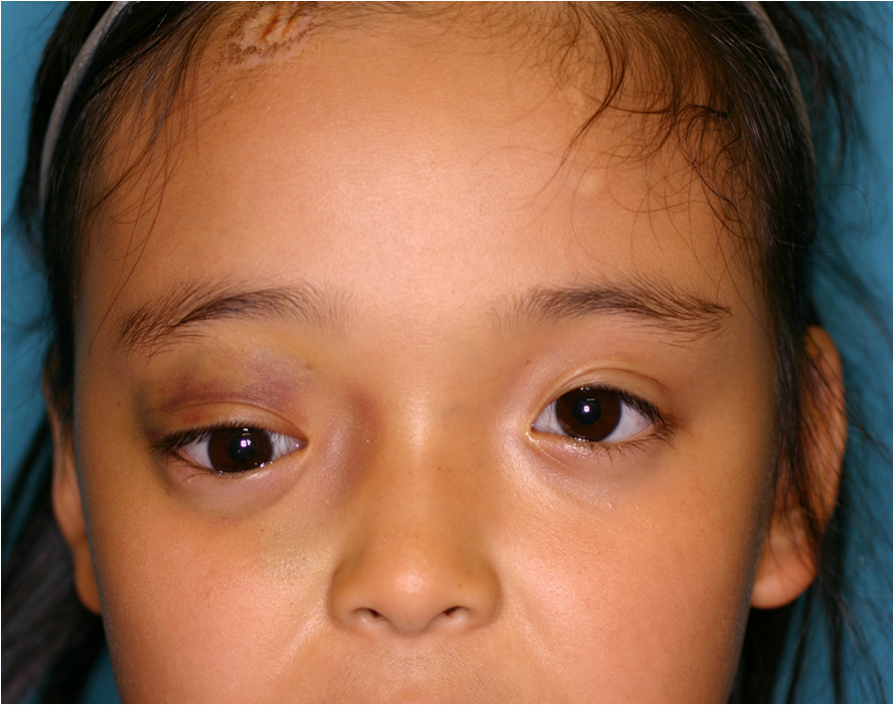
**Frontal view of the patient at the first visit.** Purpura in the right upper eyelid and dislocation of the right eyeball is observed.

At the time of her first visit to our department, her right side upper and lower palpebrae were still edematous, with non-pulsatile proptosis (Figure 1). Horizontally, her right pupil was set lower than her left, suggesting displacement or deformity of her ocular globe. The pupils were isochoric and round shaped without any pathological light reflex. While her visual acuity and intraocular pressure were within normal limits, there was a 3-mm protrusion of her right ocular globe as compared to her left globe (Additional file 1: Figure S1A). The Hess coordimetry chart showed disturbance of the upward gaze of her right eye, which was compatible with her clinical status (Additional file 2: Figure S2A, Additional file 3: Figure S3). In other words, proptosis of the right eye and diplopia were observed.

**Figure 2:**
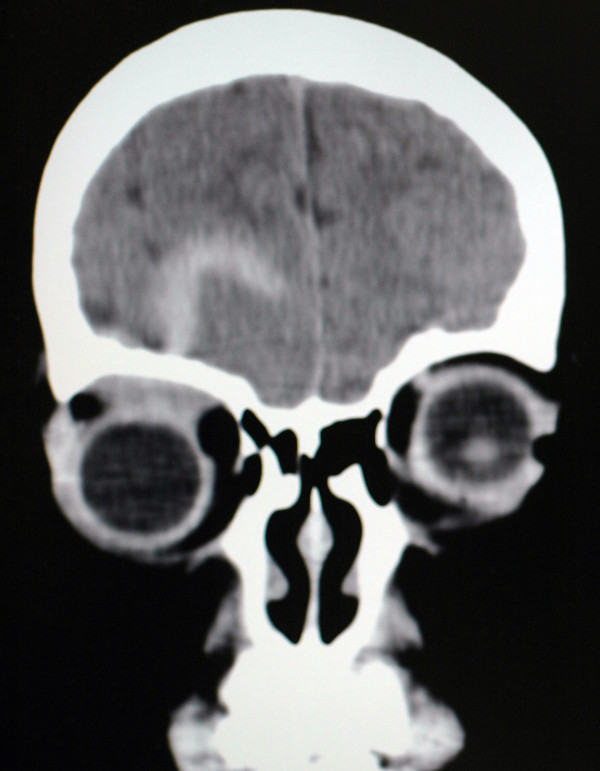
**Reconstructed coronal view of a CT image taken at the first visit.** A contrast enhanced lesion can be seen within the right frontal lobe area. A low density area is located in the right orbit, just below the upper margin.

Although plain roentgenograms indicated there was no sign of fracture, CT imaging found a right subfrontal extradural hematoma and exophthalmos of her right eye, with a low-density area also noted in the ipsilateral orbit (Figure 2). Magnetic resonance imaging (MRI) demonstrated the presence of a convex-lens-shaped space occupying lesion that was attached to the upper wall of the right orbit, and which showed isointensity to muscle in the T1-weighted image and high intensity in the T2-weighted image (Additional file 4: Figures S4A,4B). Both the clinical findings and the imaging diagnosis confirmed there was a subperiosteal hematoma in the right orbit.

**Figure 3:**
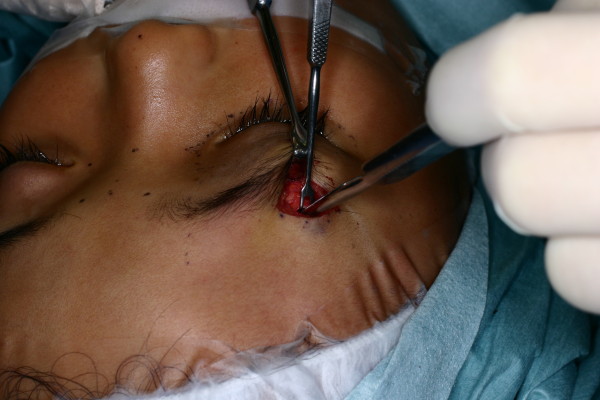
**A picture taken during the operation.** Subperiosteal approach was performed during the operation. Although the procedure was performed 14 days after the initial injury, the backflow of the irrigation saline was still clearly red.

Although the patient’s exophthalmos, disturbance of upward gaze and diplopia did not improve, there were also no changes in her central nervous system symptoms. After a neurosurgeon examined the patient, surgical treatment was prescribed and performed 14 days after her injury. An incision line was made just above the upper lateral border of her right eyebrow. After incision of the periosteum on the upper border of the orbit, the subperiosteal space was dissected towards the apex of the orbital cone (Figure 3). There was expulsion of a dark red serous discharge from the wound during the surgical procedure.

Due to continuation of the clear red discharge that was observed during irrigation of the area, a drain was placed within the subperiosteal space at the end of the procedure. After the surgery, there was improvement of both the exophthalmos and the malposition of the right ocular globe, which was in line with the lack of disturbance noted for the forced duction test.

**Figure 4:**
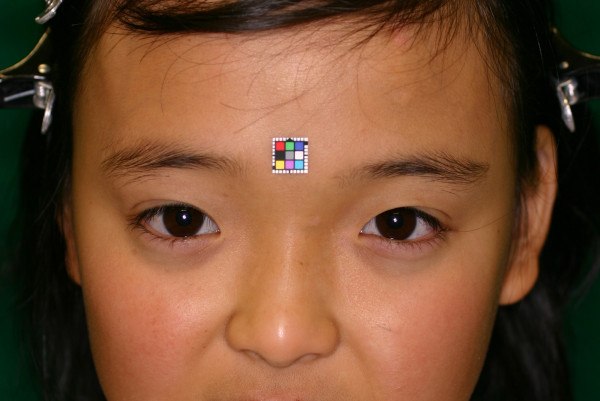
**Frontal view of the patient 2 years after treatment.** Displacement of the right eye has improved.

A Hess coordimetry chart was used to record the patient’s condition during a 4-week period after the operation. During this time, the patient showed improvement of her diplopia (Additional file 2: Figure S2B). While the eye movement and diplopia recovered, a slight asymmetry of the location of the ocular globes still remained at 60 days after the operation. There was no deficit of the central nervous system observed during the clinical course, and there was no recurrence of the subperiosteal lesion noted in the CT images on either the day after the procedure or at 6 months after the operation (Additional file 5: Figure S5). At 2 years after the surgical procedure, the patient’s ocular movement was smooth and there were no cosmetic problems (Figure 4, Additional file 1: Figure S1B, Additional file 6: Figure S6).

## Discussion

Usually, retrobulbar hematoma in the orbit and subperiosteal hematoma in the orbit are broadly interpreted as orbital hematomas. Duke-Elder et al. proposed using four general groups to classify the causes of hematoma in the orbit including: (1) trauma, (2) vascular lesion, (3) hematological disease, and (4) idiopathic [1]. Similar to the current case, they found that most of the cases they examined were due to trauma. In addition, all of the reported cases they examined were young males, with a mean age of 17 years [2,3]. The reason for this may be due to both differences in the daily activities of younger and older subjects, along with the weaker adherence of the periorbita to the orbital roof that is normally only seen in younger subjects: in other words, it is easier for posttraumatic bleeding followed by hematoma formation to occur at a younger age, as the periorbita is apt to be dissected from the orbital roof [4-6].

The common symptoms of subperiosteal hematoma in the orbit during the acute phase are exophthalmos, dislocation of the ocular globe, disturbance of the eye movement and diplopia, along with an occasional association with pain [3,6]. The patient in the current case exhibited a disturbance of upward gaze of her right eye, which might have been related to a mechanical restriction of the superior rectus muscle.

On the other hand, subperiosteal hematomas during the chronic phase have been shown to cause irreversible changes involving organization, calcification, pseudoaneurysms, or blood cysts. Therefore, all of the changes could potentially lead to exophthalmos, intraocular high pressure, corneal injury or visual acuity deficits [7,8].

The differential diagnosis of the subperiosteal hematoma of the orbit includes fracture of the orbit, internal jugular-cavernous sinus fistula and emphysema of the orbit, all of which can cause posttraumatic exophthalmos and disturbance of the eye movement. In order to make a diagnosis of subperiosteal hematoma in the orbit, it is recommended that CT or MRI be used to confirm images of distended hematoma attached to the wall of the orbit [2,9,10].

Many published studies in the literature have recommended that surgical treatments be performed for subperiosteal hematoma in the orbit, with some reporting that these treatments have resulted in a rapid recovery without sequelae [5]. In contrast, since there have been few previous reports of subperiosteal hematoma in the orbit with rapidly progressing severe symptoms such as constriction of the visual field or blindness differing from retrobulbar hematoma, other studies have stressed the importance of using observation to prevent surgical intervention-caused sequelae, such as local infection or re-bleeding [3,6]. Thus, specific treatments recommended for cases of subperiosteal hematoma of the orbit remain controversial at the present time.

In the current case, neither the proptosis, the disturbance of the ocular movement, nor the diplopia showed any improvement at 10 days after the injury. Therefore, after considering the patient’s age, the current operation was performed in an attempt to avoid any possible sequelae [11]. However, because there have been very few cases reported for subperiosteal hematoma in the orbit with an ipsilateral frontal epidural hematoma, we were initially very hesitant to perform the surgical therapy in this emergency situation [3,5-7]. After further observation and determining there were no other neurological problems in this patient, we finally decided to perform evacuation of the hematoma 2 weeks after the initial injury.

Although in this case the CT images did not indicate any signs of expansion in the subperiosteal hematoma in the orbit, we observed fresh bleeding in the backflow while irrigating the area during the surgical procedure. However, is quite likely that simply aspirating the area could have lead to this recurrence of the hematoma, as a small amount of bleeding was found around the drain on the day after the operation. Our current findings support performing surgical removal of hematomas through a small skin incision followed by the setting of a drain during the subacute stage in cases where early complications have prevented surgical treatment of subperiosteal hematomas in the orbit of younger patients. This strategy is both simple and safe, and provides a solution for patients` early complaints without the occurrence of late sequelae.

## Conclusion

We report on the clinical course of a very rare case of subperiosteal hematoma with ipsilateral frontal epidural hematoma. After ensuring there were no complications in the patient’s general status, we performed surgical treatment during the subacute phase, and the subsequent prognosis has been good. Therefore, in cases of subperiosteal hematoma in the orbit, once it has been confirmed that the patient has no other existing problems, immediate surgical therapy with a small skin incision followed by the setting of a drain is recommended in order to achieve an early resolution and avoid complications.

### Consent

Written informed consent was obtained from the patient and her parent for publication of this case report and all of the accompanying images. A copy of the written consent is available for review by the Editor-in-Chief of this journal.

## Abbreviations

CT, computed tomography; MRI, magnetic resonance imaging.

## Competing interests

The authors declare that they have no competing interests.

## Authors’ contributions

TM and MK have made substantial contributions to the acquisition of the clinical data and analysis of the previous reports in the literature. TM and JM have made decisions regarding the patient and were responsible for all treatments performed in this case. YY and KY were involved in drafting this manuscript. All authors read and approved the final manuscript.

## Pre-publication history

The pre-publication history for this paper can be accessed here:

http://www.biomedcentral.com/1471-2415/12/18/prepub

## Supplementary Material

Additional file 1 **Figure S1.** "Look-up" position at the first visit and after 2 years of treatment. Proptosis of the right eye is observed at the first visit (A). Two years after the initial treatment, proptosis of the right eye has improved (B). Click here for file

Additional file 2 **Figure S2.** Hess coordimetry. Hess coordimetry corresponds to the movement of the right eye during the patient’s first visit (A). Clinical findings also show the disturbance of the upward gaze of the right eye (Additional file 3: Figure S3). The Hess coordimetry chart recorded 4 weeks after the operation (B). This chart reveals full recovery of the eye movement, with no evidence of diplopia. The right side of these charts corresponds to the movement of the right eye. Click here for file

Additional file 3 **Figure S3.** Eye movement of the patient observed during the first visit. A severe disturbance of the right eye can be seen. Click here for file

Additional file 4 **Figure S4.** MRI taken at a previous hospital. A T1-weighted image (T1WI) shows a low intensity area in the right upper orbit (A). A T2-weighted image (T2WI) shows a high intensity area in the same portion. A high intensity area is found in the frontal cranial space, which suggests subfrontal extradural hematoma (B). Click here for file

Additional file 5 **Figure S5.** Follow-up CT images. These images show there is no remnant hematoma in the right orbit. The white line in this slice corresponds to the drain (A). There is neither a sign of recurrence of the hematoma nor any deviation of the eye noted in these CT images that were taken 6 months after the surgery (B). Click here for file

Additional file 6 **Figure S6.** Eye movement observed at 2 years after the initial surgical treatment. These photos show no sign of eye movement disturbance, which is compatible with the Hess coordimetry results (Additional file 2: Figure S2B). Click here for file
