# Supplementary figures and images for: Subacute phase treatment of subperiosteal hematoma of the orbit with epidural hematoma in the frontal cranial fossa: Case report
Source: BMC Ophthalmol. 2012 Jun 28;12:18. doi: 10.1186/1471-2415-12-18 (PMC3583151; doi:10.1186/1471-2415-12-18)

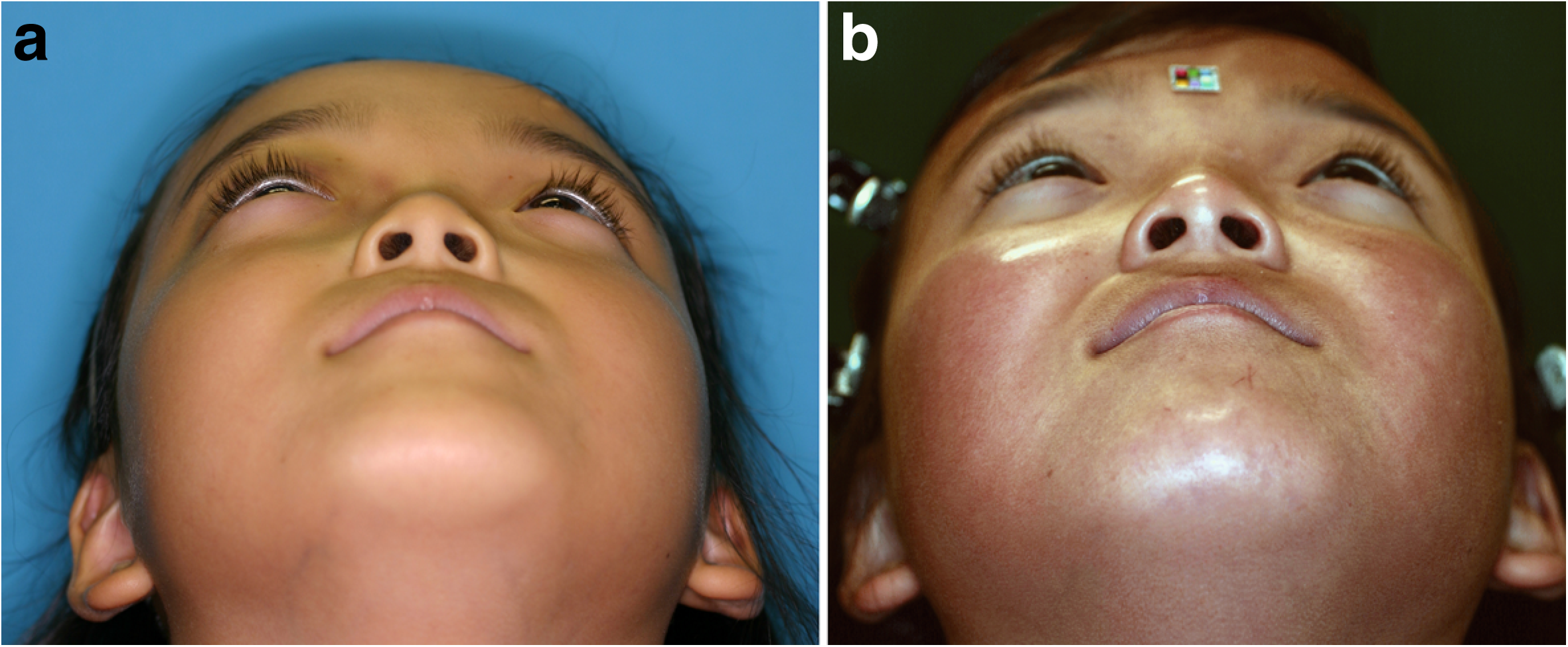

Supplement: Additional file 1 — Figure S1. "Look-up" position at the first visit and after 2 years of treatment. Proptosis of the right eye is observed at the first visit (A). Two years after the initial treatment, proptosis of the right eye has improved (B). [file 1471-2415-12-18-S1.tiff]

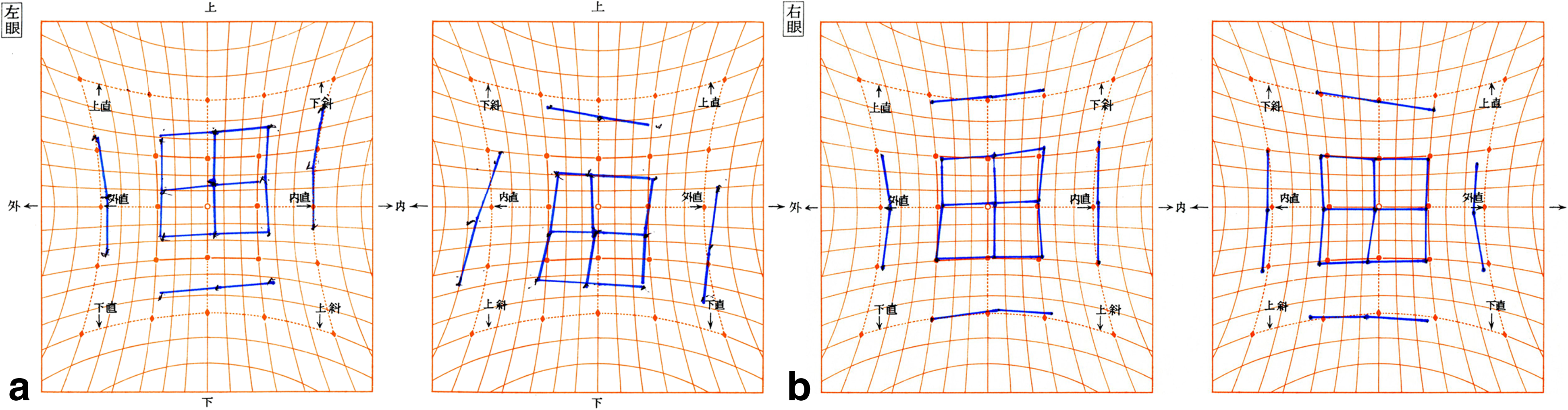

Supplement: Additional file 2 — Figure S2. Hess coordimetry. Hess coordimetry corresponds to the movement of the right eye during the patient’s first visit (A). Clinical findings also show the disturbance of the upward gaze of the right eye (Additional file 3: Figure S3). The Hess coordimetry chart recorded 4 weeks after the operation (B). This chart reveals full recovery of the eye movement, with no evidence of diplopia. The right side of these charts corresponds to the movement of the right eye. [file 1471-2415-12-18-S2.tiff]

## Slide 1
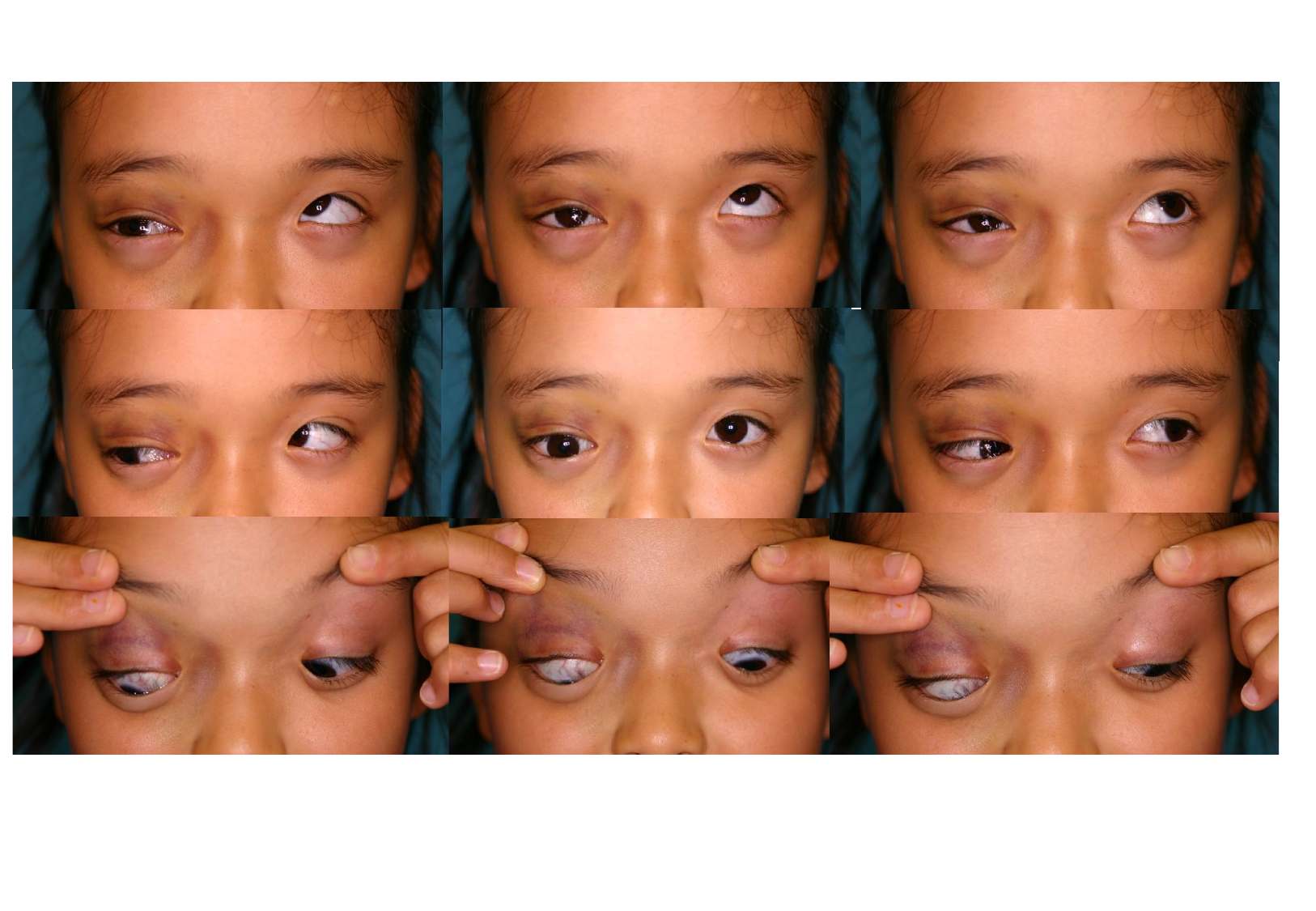

Supplement: Additional file 3 — Figure S3. Eye movement of the patient observed during the first visit. A severe disturbance of the right eye can be seen. [file 1471-2415-12-18-S3.ppt]

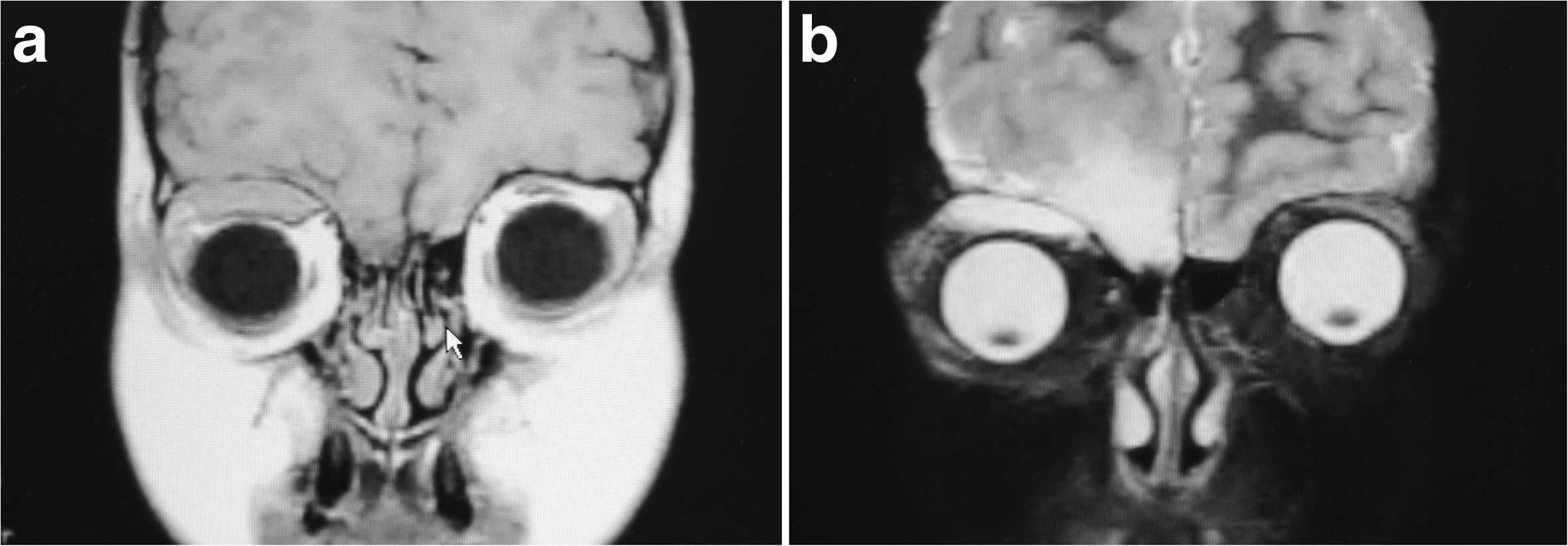

Supplement: Additional file 4 — Figure S4. MRI taken at a previous hospital. A T1-weighted image (T1WI) shows a low intensity area in the right upper orbit (A). A T2-weighted image (T2WI) shows a high intensity area in the same portion. A high intensity area is found in the frontal cranial space, which suggests subfrontal extradural hematoma (B). [file 1471-2415-12-18-S4.tiff]

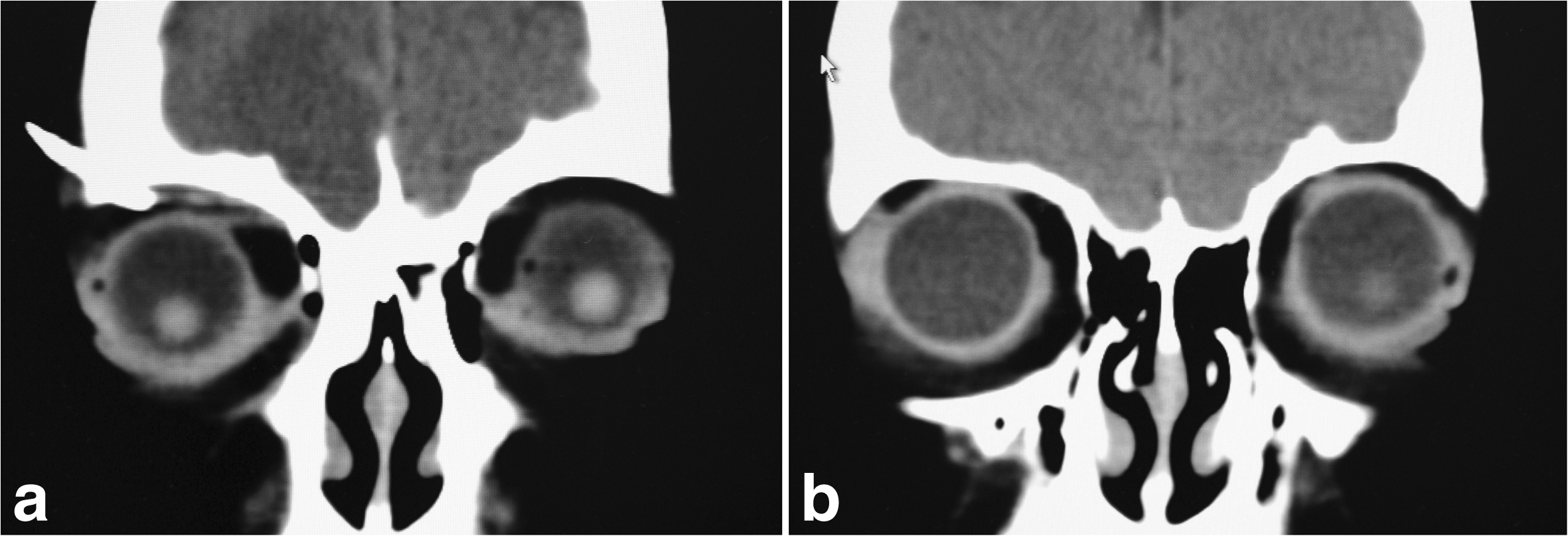

Supplement: Additional file 5 — Figure S5. Follow-up CT images. These images show there is no remnant hematoma in the right orbit. The white line in this slice corresponds to the drain (A). There is neither a sign of recurrence of the hematoma nor any deviation of the eye noted in these CT images that were taken 6 months after the surgery (B). [file 1471-2415-12-18-S5.tiff]

## Slide 1
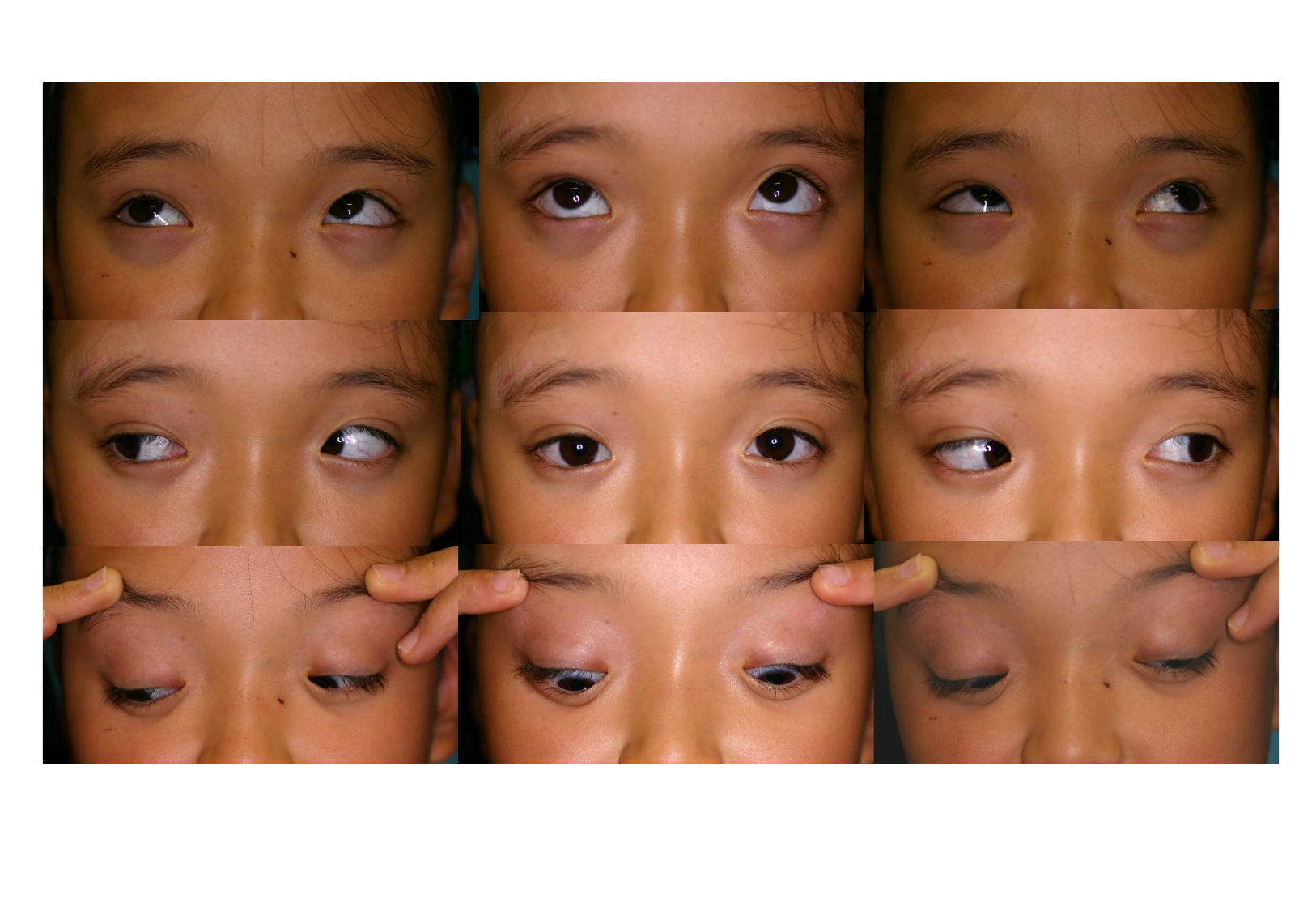

Supplement: Additional file 6 — Figure S6. Eye movement observed at 2 years after the initial surgical treatment. These photos show no sign of eye movement disturbance, which is compatible with the Hess coordimetry results (Additional file 2: Figure S2B). [file 1471-2415-12-18-S6.ppt]
